# Efficacy of Silodosin and Tadalafil monotherapy versus combination of both drugs as MET for distal ureteric stones: A prospective, double blinded, randomized clinical trial

**DOI:** 10.1080/20905998.2024.2403274

**Published:** 2024-09-16

**Authors:** Tamer Diab, Abdallah Fathi, Amr S. El-Dakhakhny, Ahmed Abou Elezz

**Affiliations:** Urology Department, Faculty of Medicine, Benha University, Benha, Egypt

**Keywords:** Silodosin, Tadalafil, medical expulsive therapy, ureteric stones

## Abstract

**Objective:**

To assess efficacy and safety of silodosin, tadalafil, versus combination of both medications as medical expulsive therapy (MET) for distal ureteric stones.

**Methods:**

This prospective, double blinded, randomized clinical trial included 128 patients aged 18 years or more irrespective of gender who are presented to emergency department or outpatient with distal ureteric stones ranging in size from 5–10 mm, randomized into three groups: Group I (*n* = 42) received Silodosin 8 mg once daily, Group II (*n* = 44) received Tadalafil 5 mg once daily, and Group III (*n* = 42) received Silodosin 8 mg combined with Tadalafil 5 mg one daily. All participants underwent thorough history-taking, routine laboratory investigations, and clinical examinations.

The primary end point included expulsion rate and time and treatment tolerance. Secondary end points were number pain episodes, need for emergency room visit, amount of analgesia, need for intervention.

**Results:**

The expulsion rate was 71.4% for silodosin, 61.4% for tadalafil and 88.1% for combined therapy (*p* = 0.018). Expulsion time was significantly shorter after combined therapy 10.4 + 3.5 days, while after silodosin 14.1 + 4 days, while 17.8 + 3.4 days after tadalafil (*p* < 0.001). The number of pain episodes, emergency room visits, and the amount of analgesia were significantly in favour of combined therapy (*p* value 0.004, 0.010, and <0.001) respectively.

**Conclusions:**

A combination of Silodosin with Tadalafil as a MET for distal ureteric stones is more effective than monotherapy with tolerable side effects.

## Introduction

Urolithiasis is a highly prevalent urinary system condition, with a lifetime prevalence of up to 15% and a high recurrence rate. Men are three times more likely to be affected than women, and the global prevalence of Urolithiasis is increasing [1]. Ureteric stones constitute approximately one-fifth of urinary tract stones, among which two-thirds located in the distal ureter [2], causing varying degrees of colicky pain and is one of the most frequent causes of emergency room visits by patients [3].

Medical expulsive therapy (MET), as a non-surgical Urolithiasis management strategy, involves treating the ureteric smooth muscles with various medications are currently the most common strategy used by urologists [4]. Among these medications are silodosin, selective alpha-1 adrenergic receptor blocker and tadalafil, a type 5 phosphodiesterase inhibitor (PDE-5 inhibitor), the rational of these medications is that tadalafil, causing ureteric smooth muscle relaxation through an action on nitric oxide-cyclic guanosine monophosphate signalling pathway of smooth muscles, resulting in increased levels of cyclic guanosine monophosphate [5]. On the other hand, silodosin, as a selective α1A-adrenoceptor antagonist inhibit ureteral contractions and facilitate stone passage with fewer side effects [6,7].

The combination of both medications might be beneficial MET for distal ureteric stones, as silodosin was reported to be most efficacious drug as MET than alfuzosin and tamsulosin [8]. Adding tadalafil to tamsulosin revealed good results for distal stone expulsion [9].

In this prospective study, our aim was to compare the efficacy of silodosin and tadalafil as monotherapy versus combination of silodosin plus tadalafil as a MET for distal ureteric stones. To our knowledge, till the time that we write this manuscript there is insufficient research evaluating the efficacy of tadalafil, Silodosin, and Silodosin plus Tadalafil as MET for lower ureteric stones. The combination of tadalafil and silodosin may result in a greater percentage of stone expulsion rate due to their efficacy with different modes of action.

## Patients and methods

This prospective, double blinded, randomized clinical trial was conducted at the Urology Department of Benha University Hospital between March 2023 and June 2024, following the institutional approval (Approval code: RC 22-3-2023) and registered on ClinicalTrials.gov (ID: NCT05789732). Written informed consent was obtained from all participants.

The study included patients aged 18 years or older, irrespective of gender, presenting to the emergency room with ureteric colic and diagnosed with distal ureteric stones ranging from 5 to 10 mm in size. Patients were excluded if they had a single kidney, renal dysfunction, urinary tract infection (UTI), bilateral or multiple ureteric stones, severe, intractable pain requiring emergency intervention, marked hydronephrosis, ischemic heart disease, pregnancy, complicated hypertension, a history of ureteral surgery or any urologic anomalies, congestive cardiac failure, or concurrent administration of calcium channel blockers or nitrates. Also, patients with a previous history of successful stone passage or endoscopic management were excluded from the study to eliminate any factor influence subsequent stone passage other than our medications effect.

## Baseline investigations

To prepare the study population, the following baseline investigations were performed:

## Medical history and physical examination

A detailed medical history was taken, including information on previous episodes of Urolithiasis, comorbidities, medication use, and allergy history. A comprehensive physical examination was conducted, focusing on the abdomen and genitourinary system.

## Imaging studies

A non-contrast Computed Tomography (CT) scan of the abdomen and pelvis was performed to confirm the presence, size, and location of distal ureteric stones, but stone density (HU) is not a reliable predictor of stone expulsion, so it was not been adopted as a criterion in statistical analysis [10]. An ultrasound of the kidneys and bladder was also conducted to assess for hydronephrosis and other renal anomalies.

## Laboratory tests

A complete blood count (CBC) was performed to evaluate hemoglobin levels, white blood cell count, and platelet count. Serum creatinine and estimated glomerular filtration rate (eGFR) were measured to assess renal function. Serum electrolytes, including sodium, potassium, calcium, and phosphate levels, were analyzed. Urinalysis was conducted to check for the presence of blood, leukocytes, nitrites, and other abnormalities. A urine culture was performed to rule out urinary tract infections (UTI).

## Randomization and blinding

Patients were randomized using a computer-generated randomization table into three groups: Group I (*n* = 48) received Silodosin 8 mg once daily, Group II (*n* = 48) received Tadalafil 5 mg once daily and Group III (*n* = 48) received Silodosin 8 mg combined with Tadalafil 5 mg once daily. Diclofenac sodium 50 mg tablets were advised orally as needed for mild to moderate pain, and 100 mg injections were administered during severe episodes, and all were calculated.

The study medications were identical in appearance (color, shape, and packaging) and labeled with unique code numbers by a third-party pharmacist not involved in the study. Neither the participants nor the healthcare providers administering the treatments were aware of the group assignments, ensuring both patient and clinician blinding. Outcome assessors, including those performing follow-up imaging and evaluating adverse events, were blinded to the treatment allocation and recorded and analyzed the data without knowing which group the participants were assigned to.

Participants were given detailed instructions on how to take their medications and were monitored to ensure adherence to the blinding protocol, and they were instructed not to discuss their medication details with the healthcare providers or other study participants.

## Pain assessment

The severity of pain was assessed using the Visual Analog Scale (VAS) [11], a validated tool for pain measurement. Patients were asked to rate their pain on a scale from 0 to 10, where 0 represented no pain and 10 represented the worst possible pain. This assessment was conducted at baseline, during follow-up visits, and at the time of any pain episodes requiring analgesic intervention. Pain episodes were recorded by the patients in a daily pain diary, noting each occurrence of pain throughout the study duration. The total number of pain episodes reported was analyzed for the entire study period.

## Fatigue assessment

Patients’ fatigue was assessed using the Fatigue Severity Scale (FSS) [12], a validated and widely used tool to measure the impact of fatigue on daily functioning. The FSS consists of nine statements that patients rate on a scale from 1 to 7, where 1 indicates strong disagreement and 7 indicates strong agreement. The total score is calculated by averaging the scores of the nine items. This assessment was performed at baseline and during follow-up visits to monitor changes in fatigue levels throughout the study period.

## Follow-up and outcome measures

Patients were instructed to filter their urine or use a urinal to detect any passing stones. Follow-up was conducted with a CT scan after stone passage (which detected by stone captured or visualized by the patient during urination) or after 4 weeks on medication without stone passage. Primary end point included expulsion rate and time and treatment tolerance. Secondary end points were number of pain episodes, need for emergency room visit, amount of analgesia, need for intervention. Shifting to ureteroscopic stone retrieval due to intractable pain, progressive hydronephrosis, and lack of progress after 4 weeks of treatment or no conclusive evidence that long-term use of these drugs could alleviate obstructive uropathy symptoms or improve stone expulsion rates.

## Sample size estimation

The required sample size was determined using G*power software (v.3.1.9.7; Heinrich-Heine-Universität Düsseldorf, Düsseldorf, Germany) [13]. Considering an anticipated medium effect size (d = 0.5) regarding the expulsion time between the groups being studied. To identify such a medium effect size, a minimum of 120 participants (40 patients per group) was necessary. To account for potential attrition during follow-up, the sample size was increased to 132 (43 participants in each group). The significance level (alpha) and statistical power were set at 0.05 and 0.8, respectively.

## Statistical analysis

Data management and statistical analysis were conducted using SPSS version 28 (IBM, Armonk, New York, United States). The normality distribution of the data was determined using histograms and the Shapiro-Wilks test. Parametric and non-parametric data analyses were applied as appropriate. Mann-Whitney Test and Kruskal-Wallis Test were used as appropriate. ANOVA test was used for comparing expulsion time in groups with Bonferroni post hoc pairwise comparison comparing silodosin and tadalafil versus the combined group. Binary logistic regression was run and the variables entered in the analysis were the stone size and groups with the combined therapy group entered as the reference category to the other groups to examine the predictors of success of MET. All statistical tests were two-tailed and a P-value <0.05 was considered statistically significant.

## Results

**Figure 1.**
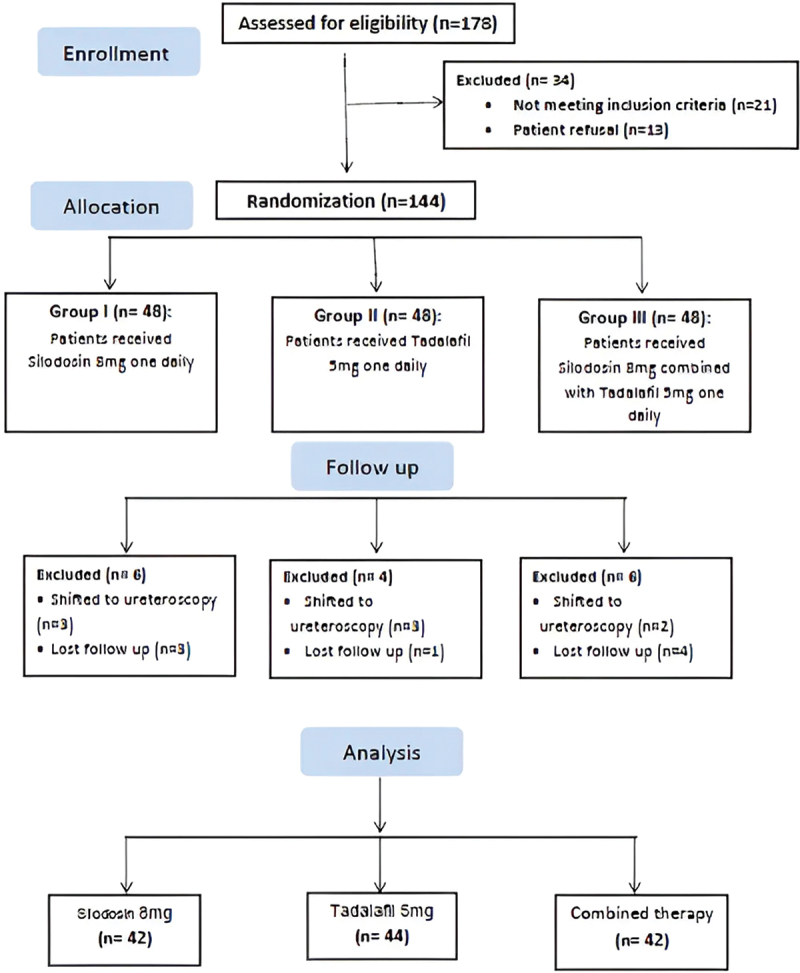
CONSORT flow chart of the studied groups.

A total of 178 cases were screened for eligibility, of which 21 cases did not meet the inclusion criteria and 13 cases declined to participate. The remaining eligible 144 cases were randomized into three groups; each group contain 48 patient and all cases received the allocated treatments. Another 16 cases were excluded from the study in the three groups, 8 patients had attack of sever refractory colic and shifted to ureteroscopy, other 8 patients were lost follow up. The remaining 128 cases in three groups received the allocated medications and complete follow up till the end of 4 weeks, who were available for statistical analysis Figure 1.

Forty-two cases received silodosin 8 mg once daily per oral, 44 received tadalafil 5 mg once daily per oral, and 42 patients received a combination of both drugs. Patients’ demographics and outcome are presented in (Table 1).

**Table 1.** Patients’ demographics and outcome in the studied groups.

		Silodosin N = 42	Tadalafil N = 44	Combined N = 42	Total	P value
Age		39.2 ± 7.9	35.7 ± 6.8	37.5 ± 7.7	37.4 ± 7.6 (24–45)	0.094***
Gender, N (%)	Male	26 (61.9)	29 (65.9)	28 (66.7)	83 (64.8)	0.886*
	Female	16 (38.1)	15 (34.1)	14 (33.3)	45 (35.2)	
BMI		23.9 ± 2.2	24.1 ± 2.4	23.4 ± 2.5		0.353***
Laterality	Right	23 (54.8)	24 (54.5)	26 (61.9)	73 (57)	0.738*
	Left	19 (45.2)	20 (45.5)	16 (38.1)	55 (43)	
Stone size (mm) Median (range)		6.5 (5.2–9)	6.5 (5.2–9)	6.5 (5.2–9)		0.987**
Expulsion rate, N (%)		30 (71.4)	27 (61.4)	37 (88.1)	94 (73.4)	0.018*
Expulsion time (days) Mean +SD (range)		14.1 ± 4 (8–23)	18 ± 3.3 (13–25)	10.4 ± 3.5(6–22)		<0.001***
Expelled size (mm) Median (range)		6.4 (5.2–9)	6 (5.2–8.5)	6.5 (5.2–9)		0.611**
Pain episodes		1.1 ± 1.2	1.6 ± 0.9	0.9 ± 0.9		0.004***
Hospital visits		0.8 ± 1	1 ± 0.9	0.4 ± 0.7		0.010***
Analgesia dose (mg)		508.9 ± 480.5	612.5 ± 334.9	276.8 ± 271.4		<0.001***
Side effects		N (%)	N (%)	N (%)		
	Ret. Ejaculation	22 (52.4)	1(2.3)	23 (52.3)	64 (50)	
	Backache	1 (2.4)	4 (9.1)	3 (7.1)	7 (5.5)	
	Dizziness	3 (7.1)	4 (9.1)	4 (9.5)	11 (8.6)	
	Headache	6 (14.3)	9 (20.5)	5 (11.9)	20 (15.6)	
	Orthostatic hypotension	2 (4.8)	2 (4.5)	3 (7.1)	7 (5.5)	
	Runny nose	1 (2.4)	1 (2.3)	2 (4.8)	4 (3.1)	

*Chi square test. **Kruskal-Wallis Test. ***ANOVA test.

The study groups were comparable regarding age, gender, BMI, laterality, and stone size. Median stone size in all studied groups was 6.5 mm and range from 5.2 to 9 mm. Expelled median stone size in all groups was 6.35 mm (range 5.2–9 mm), while in failed MET median stone size was 7.6 mm (5.4–9 mm) with significant difference (*p* value < 0.001, Mann-Whitney Test), data are not presented in the table.

Median stone size expelled in combined therapy group was 6.5 mm larger than other groups, yet this was statistically insignificant (*p* value 0.611, Kruskal-Wallis Test).

Expulsion rate was significantly higher with combined therapy group (*p* value 0.018). Expulsion time was significantly shorter with combined therapy group, mean +SD was 10.4 + 3.5 and range from 6 to 22 days. The combined MET group showed significant shorter expulsion time than the monotherapy groups, Bonferroni post hoc pairwise comparison revealed significant mean difference for combined therapy versus silodosin and tadalafil, 3.7 and −7.7 days respectively (*p* value < 0.001 for each). Additionally, silodosin group showed significant shorter expulsion time versus tadalafil with mean difference −4 and *p* value < 0.001.

Pairwise comparison with significant ANOVA test regarding pain episodes, the significant difference was only found between combined therapy and tadalafil (*p* value 0.004). Hospital visits with combined therapy was significantly lower than with tadalafil (*p* value 0.009). The need for analgesia with combined therapy was significantly lower than with silodosin (*p* value 0.015) and with tadalafil (*p* value < 0.001). These results indicating that combined therapy group had significant fewer pain episodes and lower hospital visits and need for analgesia. Fatigue Severity Scale was assessed, but no statistical significant differences were observed between groups.

Binary logistic regression was run and the variables entered in the analysis were the stone size and groups where the combined therapy group entered as the reference category to the other groups to examine the predictors of success of MET. It was found that the odds ratio for stone size was 0.292 (inverting this odds ratio for easier interpretation), indicates that for each one-point (1 mm) increase in stone size there was a 3.4 folds risk that the stone would not be expelled with MET. Group is tested as a whole and then silodosin and tadalafil compared to the reference category which is combined therapy. For the theory dummy variable, the 0.230 odds ratio for Silodosin means that the odds of success of MET are only 0.230 times those of combined therapy. Inverted odds ratios for the dummy variables coding for easy interpretation indicated that the odds of MET success for the combined treatment were 4.3 times higher than that for the silodosin alone and 6.8 times higher than that for the tadalafil alone (Table 2).

**Table 2.** Binary coefficient of regression analysis for studied groups and stone size.

		B	P value	Exp(B) Odds ratio	95% C.I.for EXP(B)	
					Lower	Upper
Step 1^a^	Group		0.017			
	Group (1)Silodosin	−1.469	0.031	0.230	.060	.877
	Group (2)Tadalafil	−1.923	0.005	0.146	.039	.553
	Stone size	−1.232	<0.001	0.292	.178	.477

## Discussion

MET is a highly recommended treatment method for increasing stone expulsion rate due to its effectiveness in reducing symptoms and facilitating the passage of stones [14] To reduce the need for analgesics and improve stone passage, PDE5-Is, calcium channel blockers (CCBs), and alpha-blockers are commonly used in MET [15]. *Gratzke* et al. [5], revealed that PDE inhibitors have a role in relaxing ureteric muscles in the order of tadalafil> vardenafil > sildenafil, which influenced our decision to use tadalafil. Tadalafil’s activity is not affected by meals and has the longest duration of action among currently available PDE-5 drugs (36 hours and a half-life of 17.5 hours). In comparison to sildenafil, tadalafil is more selective for PDE-5 receptors than PDE-6 receptors, which are located in the retina, making visual issues less likely to occur [16,17].

We administered a lower effective daily dose of tadalafil (5 mg) to minimize side effects. *Kloner et al*. [18], determined that the combination of tamsulosin and tadalafil was safe, and *Bechara* et al. [19], demonstrated its efficacy in managing lower urinary tract symptoms. Therefore, we are looking forward to evaluate the safety and efficacy of adding tadalafil to a more potent alpha-adrenergic receptor blocker silodosin in comparison to their effect alone for lower ureteric stones. The combination of tadalafil and silodosin may result in a greater percentage of stone expulsion rate due to their different modes of action as silodosin works as an a1-ARS blocker, while tadalafil functions as a PDE5 inhibitor. Thus, their combination could potentially enhance stone expulsion [20].

Our findings showed that the expulsion rate was significantly higher in groups III and I compared to group II (37 (88.1%), 30 (71.4%), and 27 (61.4%) respectively) and was significantly higher with combined group III compared to silodosin group I (*p* = 0.018). In line with our findings, *Samir* et al. [21], conducted a study in which 90 cases were randomly assigned to three groups. Group I received silodosin 8 mg once per day, Group II received vardenafil 5 mg once per day, and Group III received a combination of both once per day which had a significantly higher rate of stone expulsion than the other groups that received only silodosin or vardenafil (90% vs. 76.7% vs. 60%, *p* = 0.025). In the same direction where recent literature concluded Silodosin – Tadalafil and Silodosin – Vardenafil combinations are effective for facilitating the expulsion of distal ureteric stones, but Silodosin – Tadalafil combination is more tolerable and associated with a significantly lower incidence of adverse effects [22].

According to expulsion time, we found that it was significantly shorter mean expulsion time in groups III and I compared to group II (10.4 + 3.5(6–22), 14.1 + 4 (8–23), 18 + 3.3 (13–25), respectively *p*= <0.001). Results convergent to Ours by *Girish* et al. [23], found that combination group exhibited shorter expulsion time compared to the tadalafil & tamsulosin groups separately.

In our present study, the number of pain episodes was significantly lower in group III (0.9 + 0.9) compared to groups I and II a (1.1 + 1.2 and 1.6 + 0.9 *p* = 0.004). In addition, it was significantly higher in group II compared to groups I. The number of hospital visits was significantly higher in groups II and I compared to group III (*p* = 0.010). Furthermore, the amount of analgesia was higher in groups II and I compared to group III (*p* < 0.001). *Rahman* et al. [19]. found that combination of silodosin and tadalafil considerably reduced the frequency of pain episodes, which could be attributed to the distinct mechanisms of action of the two medications. Tadalafil reduces pain episodes by lowering the intraluminal ureteric pressure and reducing the amplitude and frequency of ureteric phasic contractions, while silodosin only inhibits the C fibers [24].

In terms of side effects, retrograde ejaculation was significantly more common in groups I and III compared to groups II (52.4%, 52.3%, and 2.3% respectively). Other side effects, including dizziness, headache, backache, orthostatic hypotension, and runny nose, were comparable among the studied groups and all of these adverse effects were mild and well-tolerated. No participants were excluded from the study due to the presence of adverse effects. In addition, the study by *Samir* et al. [21]. showed that headache, dizziness, and orthostatic hypotension were comparable among the studied groups. This finding can be attributed to the young age of the study population. However, retrograde ejaculation was significantly higher in groups treated with silodosin and the combination therapy (86.7%) compared to vardenafil alone (0%).

Also, *Goyal* et al. [25]. found that there was no significant difference in adverse drug effects among the studied groups, except for the tamsulosin group, which had a significantly higher incidence of retrograde ejaculation. Although the findings of the meta-analysis did not support replacing alpha blockers with tadalafil, several studies evaluating the efficacy of tadalafil in stone expulsion have attracted the attention of urologists towards using it as a MET. These studies suggest that combining of alpha blockers with tadalafil in MET may reduce the need for minimally invasive procedures and Shock Wave Lithotripsy (SWL) therapy [26,27].

Finally at the end of 4 weeks, 34 patients with no stones passage in different groups are followed up by CTUT scan to confirm presence of stone, which was fond in 29 patients and were shifted to be managed by semi rigid ureteroscopy with or without DJ fixation. The remaining five patients (2 in Sildosin and 3 in combined group) were passing stone without them realizing it.

Our study has some limitations despite being conducted with prospective randomization; it was conducted at a single center. Therefore, further evaluation is required on a larger scale at multiple centers to establish the role of silodosin and tadalafil as MET for lower ureteric stones. Additionally, patients with no stone passage complete the duration of our study (4 weeks) with more pain episodes and analgesics.

## Conclusions

The combination of tadalafil with silodosin as a MET has shown to be more effective and safer compared to using either medication as a monotherapy. The combination treatment was found to significantly increase the rate of lower ureteric stone expulsion, while also reducing the time of expulsion and pain episodes when compared to silodosin or tadalafil alone.

## Registration

The study was registered on ClinicalTrials.gov No (ID: NCT05789732).
